# From surgical treatment to psychiatric progress in refractory obsessive–compulsive disorder: A case report

**DOI:** 10.1002/pcn5.70201

**Published:** 2025-09-03

**Authors:** Shotaro Fujiwara, Yasushi Okamura, Hitomi Wake, Hideaki Tanami, Takuto Ishida, Masafumi Mizuno

**Affiliations:** ^1^ Department of Psychiatry Tokyo Metropolitan Matsuzawa Hospital Tokyo Japan; ^2^ Department of Surgery Tokyo Metropolitan Matsuzawa Hospital Tokyo Japan; ^3^ Department of Internal Medicine Tokyo Metropolitan Matsuzawa Hospital Tokyo Japan

**Keywords:** behavior therapy, laparoscopy, obsessive–compulsive disorder, rectal prolapse, self‐injurious behavior

## Abstract

**Background:**

Obsessive–compulsive disorder (OCD) can cause physical complications, and psychiatric treatment sometimes improves these complications. However, it remains unclear whether managing a physical complication can contribute to the improvement of psychiatric symptoms or may alter the trajectory of psychiatric treatment.

**Case Presentation:**

We report on a woman in her 50s with severe, long‐standing, treatment‐resistant OCD centered on contamination fears and compulsive defecation rituals. She rarely sought psychiatric help, and her symptoms worsened. Her compulsions led to rectal prolapse and fecal incontinence, which in turn exacerbated her OCD in a vicious cycle. After laparoscopic rectopexy resolved her incontinence, a marked reduction in repetitive cleaning behaviors occurred, including decreased time spent in the toilet and reduced toilet paper use. The physical improvement was followed by psychiatric engagement, regular outpatient visits, and subsequent therapeutic progress.

**Conclusion:**

This case illustrates that a physical intervention could do more than alleviate somatic distress; it could act as a catalyst for psychiatric care. By breaking the cycle between a physical symptom and a compulsive behaviors, the surgical treatment created a crucial opening for establishing trust and motivation. This highlights the importance of integrated, cross‐disciplinary collaboration in managing complex OCD cases where somatic and psychiatric symptoms are deeply intertwined.

## BACKGROUND

Obsessive–compulsive disorder (OCD) is frequently associated with physical comorbidities,^1^, ^2^, ^3^, ^4^, ^5^ which can be severe.^6^, ^7^, ^8^, ^9^, ^10^ Past reports suggest that bowel obsessions^11^, ^12^ can cause bowel complications, including rectal bleeding and rectal prolapse. In patients with such symptoms, while parallel treatment of the physical and psychiatric symptoms is recommended,^4^, ^13^, ^14^ successful psychiatric treatment of OCD can subsequently resolve the associated physical complications.^9^, ^10^ However, the reverse pathway remains unclear: whether the treatment of a physical complication can improve OCD symptoms. This approach is often challenging, as without psychiatric recovery, the risk of symptom recurrence persists.^8^, ^15^ Surgery is sometimes postponed or not considered until mental symptoms improve, due to the high risk of recurrence,^16^, ^17^, ^18^ thus, there are few studies showing that prior physical treatment has a positive effect on psychiatric treatment. Herein, we report a case of severe, treatment‐refractory OCD with symptoms including repetitive cleaning, which were tightly linked to recurrent prolapse. A laparoscopic rectopexy was followed by a reduction in repetitive cleaning behaviors and the patient's subsequent engagement in psychiatric treatment.

## CASE PRESENTATION

A woman in her 50s with more than a 30‐year history of OCD presented with rectal prolapse. Her OCD was characterized by severe fears of contamination and compulsive cleaning behaviors. Her obsessions led to extreme cleaning rituals centered on defecation; she strained excessively for a sense of complete evacuation, often spending more than 5 h on the toilet and using up to 12 rolls of toilet paper daily. Despite over 20 years of pharmacotherapy, her insight remained limited, and she persistently refused all psychiatric hospital visits, psychiatric consultations, and other welfare services, and remained housebound. Her mother attended psychiatric outpatient visits on her behalf to obtain medication, which she reluctantly continued under family persuasion but refused to have adjusted. Her severe compulsions occupied most of the day, including not only prolonged and frequent showering and handwashing to cleanse herself, but also repeated cleaning of the toilet and bathroom, which she felt had become contaminated. She was unable to use public toilets, which further limited her ability to leave home. Having never experienced psychiatric treatment sessions, she perceived little value in attending them. Notably, her motivation for this visit was purely physical, underscoring her disengagement from psychiatric care.

The patient's compulsions caused her to dilate her anal sphincter excessively, thereby leading to a rectal prolapse and fecal incontinence. These in turn further intensified her contamination fear. Caught in this vicious cycle, she repeatedly required her family, day and night, to check for fecal soiling and clean her if necessary. In year X‐6, after her family reported the rectal prolapse, her primary psychiatrist referred her to both the psychiatry and surgery departments at the study center. The patient initially underwent a perineal procedure with limited psychiatric intervention. This failed to resolve the fecal incontinence and underlying psychiatric issues, as her compulsive straining persisted, leading to a recurrence of the prolapse by year X‐3. The recurrence was confirmed in year X. The clinical team recognized that the persistent fecal incontinence was exacerbating her psychiatric symptoms and creating a vicious cycle that worsened the prolapse. They therefore opted for a different strategy. This led to a second surgical intervention: a laparoscopic rectopexy with polypropylene mesh (PROLENE Mesh, ETHICON, Johnson & Johnson). This approach, while technically more demanding, is associated with a lower risk of recurrence and a lower incidence of postoperative fecal incontinence.^19^, ^20^, ^21^ Prompted by ongoing contact with her psychiatrist and strong recommendations from her surgeon, with whom she had established trust, she reluctantly agreed to concurrent psychiatric treatment during this surgical admission, which decreased her psychological resistance toward psychiatry. Postoperatively, she reported feeling “less dirty” because her fecal incontinence had resolved. Her cleaning behaviors diminished; her time on the toilet decreased from over 5 to 2 h, and her daily toilet paper use fell from 12 rolls to 2. Although these reduced levels still represented significant residual compulsive rituals, she was no longer reluctant to see a psychiatrist.

The partial reduction in her cleaning behaviors was a critical juncture. It appeared to foster the patient's trust in medical care and created a therapeutic window for intensive psychiatric intervention. The psychiatric team utilized this opening by providing psychoeducation and initiating cognitive‐behavioral therapy (CBT) concurrently with pharmacotherapy. Her attitude changed significantly: she developed insight and motivation, and began attending appointments regularly. The subsequent 1.5 years of this sustained therapy resulted in measurable improvement: her Global Assessment of Functioning (GAF) score increased from 30 to 45, and her Yale‐Brown Obsessive Compulsive Scale (Y‐BOCS) score gradually declined from 38 to 34.

## DISCUSSION AND CONCLUSION

This case offers a new perspective on the physical management of somatic symptoms driven by psychopathology. Past reports warned that physical interventions targeting psychiatric‐driven symptoms may have limited effectiveness.^8^, ^15^, ^22^ This was reflected in the patient's first perineal surgery without a collaborative approach, which was followed by recurrence. In contrast, the second intervention illustrated how a synergistic, collaborative approach addressing both the physical condition and its psychiatric drivers could be associated with long‐term improvement. In this case, the successful physical intervention appeared to play an instrumental role in building the patient's trust in medical care, thereby fostering the motivation necessary for psychiatric engagement.

While psychiatric care was essential for long‐term improvement, in cases such as the present one, the surgical intervention generally constituted the initial treatment because the patient's primary concern was physical in nature. Consistent with models of OCD in which compulsions are regarded as repetitive behaviors triggered by preceding stimuli, such as uncomfortable physical sensations,^23^, ^24^, ^25^, ^26^ her incontinence and physical discomfort appeared to exacerbate her symptoms. Although it was not a treatment for OCD itself, the surgery reduced the perpetuating triggers and was followed by a marked reduction in the patient's repetitive cleaning behaviors. In addition, this improvement appeared to lessen her psychological burden and increased her functional capacity to engage in outpatient care. This outcome is consistent with a previous case report suggesting that attenuating somatic triggers may contribute to the reduction of compulsions.^27^ Importantly, the surgical success itself also had a profound psychological effect. For this patient, who had previously been reluctant to engage in psychiatric care, the success of the physical intervention played a pivotal role in building trust, which in turn prompted her to accept psychiatric treatment. This behavioral change is consistent with reports that interventions outside the psychiatric framework may contribute to building a therapeutic alliance.^28^, ^29^, ^30^ Thus, the surgery appeared to serve not merely as a physical treatment but also as an important step in building trust and facilitating access to comprehensive treatment in the present case.

Although this is a single case, the present report may offer a hypothesis for future investigation. In patients whose repetitive behaviors are directly driven by a physical problem, targeted physical intervention might facilitate subsequent psychiatric engagement. Moreover, when integrated with psychiatric care, physical treatment may confer benefits beyond its direct physical effects in the management of complex cases in which psychiatric and somatic symptoms are closely interrelated.

It should be noted that this single case report cannot establish a causal relationship between the surgical treatment and the subsequent psychiatric improvement. Other factors—such as the natural course of illness, and intensive clinical attention during hospitalization—may have influenced the observed outcome. The possible mechanism of recovery in the present case may apply only to a small subset of patients with significant physical comorbidities. These preliminary observations warrant cautious interpretation and confirmation through larger, controlled studies.

In conclusion, this case suggests that somatic interventions may play a role not only as physical treatments, but also as potential catalysts for psychiatric engagement in patients otherwise resistant to treatment. Further research is clearly warranted to explore collaborative care approaches for OCD patients whose symptoms are triggered by physical stimuli.

## AUTHOR CONTRIBUTIONS

Shotaro Fujiwara, Yasushi Okamura, Hitomi Wake, and Hideaki Tanami acquired case data. Shotaro Fujiwara wrote the first draft of the manuscript with Yasushi Okamura's and Hitomi Wake's support. Hideaki Tanami, Takuto Ishida, and Masafumi Mizuno supervised and critically revised the manuscript. All authors have contributed to and approved the final manuscript.

## CONFLICT OF INTEREST STATEMENT

The authors declare no conflicts of interest.

## ETHICS APPROVAL STATEMENT

This case report was conducted in accordance with ethical guidelines for case reports of the Japanese Society of Psychiatry and Neurology. Written consent from the patient was obtained for the reporting of this case.

## PATIENT CONSENT STATEMENT

Written consent from the patient was obtained for the reporting of this case.

## CLINICAL TRIAL REGISTRATION

N/A.

## Data Availability

The data that support the findings of this study are available on request from the corresponding author. The data are not publicly available due to privacy or ethical restrictions.
